# Initial experience of a novel method for electrical isolation of the superior vena cava using cryoballoon in patients with atrial fibrillation

**DOI:** 10.1002/clc.23947

**Published:** 2022-11-20

**Authors:** Changjian Lin, Yangyang Bao, Yun Xie, Yue Wei, Qingzhi Luo, Tianyou Ling, Ning Zhang, Qi Jin, Wenqi Pan, Yucai Xie, Liqun Wu

**Affiliations:** ^1^ Department of cardiovascular medicine, Ruijin Hospital Shanghai Jiao Tong University School of Medicine Shanghai China

**Keywords:** atrial fibrillation, phrenic nerve, pulmonary vein isolation, second‐generation cryoballoon, sinus node, superior vena cava isolation

## Abstract

**Background:**

Damage to the sinus node (SN) has been described as a potential complication of superior vena cava (SVC) isolation. There have been reports of permanent SN injury requiring pacemaker implantation during isolation of the SVC.

**Hypothesis:**

It is safe and effective to isolate SVC with the second‐generation 28‐mm cryoballoon by using a novel method.

**Methods:**

Forty‐three patients (including six redo cases) with SVC‐related atrial fibrillation (AF) from a consecutive series of 650 patients who underwent cryoballoon ablation were included. After pulmonary vein isolation was achieved, if the SVC trigger was identified, the SVC was electrically isolated using the cryoballoon. First, the cryoballoon was inflated in the right atrium (RA) and advanced towards the SVC‐RA junction. After total occlusion was confirmed by dye injection with total retention of contrast in the SVC, the SVC‐RA junction was determined. Next, the cryoballoon was deflated, advanced into SVC, then reinflated, and pulled back gently. The equatorial band of the cryoballoon was then set slightly (4.32 ± 0.71 mm) above the SVC‐RA junction for isolation of the SVC.

**Results:**

Real‐time SVC potential was observed in all patients during ablation. The mean time to isolation was 24.5 ± 10.7 s. The SVC was successfully isolated in all patients. The mean number of freeze cycles was 2.5 ± 1.4 per patient, and the mean ablation time was 99.8 ± 22.7 s. A transient phrenic nerve (PN) injury occurred in one patient (2.33%). There were no SN injuries. Freedom from AF rates at 6 and 12 months was 97.7% and 93.0%, respectively.

**Conclusions:**

This novel method for SVC isolation using the cryoballoon is safe and feasible when the SVC driver during AF is determined and could avoid SN injury. PN function should still be carefully monitored during an SVC isolation procedure.

## 1 INTRODUCTION

Pulmonary vein isolation (PVI) is the cornerstone in the ablation of atrial fibrillation (AF) and eliminates the majority of triggers for the commencement and maintenance of AF.^1^ However, abnormal ectopic firing arising from regions other than the pulmonary vein (PV) can also trigger and maintain AF.^2^ The superior vena cava (SVC) is one of the most common non‐PV triggers of AF; therefore, it is an attractive target for ablation to maintain normal sinus rhythm (SR).^3^ Electrical isolation of the SVC by the traditional means of radiofrequency ablation (RFA) is associated with an improved outcome in terms of freedom from AF.^4^, ^5^ Recently, there have been some reports on the isolation of the SVC by cryoballoon ablation.^6^, ^7^ However, SVC isolation using a cryoballoon can be challenging because of the vicinity of the sinus node (SN) and phrenic nerve (PN). There have been reports of permanent SN injury requiring pacemaker implantation during isolation of the SVC. The aim of this study was to evaluate the safety and efficacy of a novel method for SVC isolation using a second‐generation 28‐mm cryoballoon.

## 2 METHODS

### 2.1 Study population

This study evaluated 650 consecutive patients with AF who underwent cryoballoon ablation at Shanghai Ruijin Hospital between July 2019 and July 2021. Forty‐three patients (6.62%, including six redo cases) with AF found to be originating from SVC foci during the procedure were included in the final analysis. All patients provided written informed consent before the cryoballoon ablation procedure. The study was approved by the Institutional Review Board and performed in accordance with the principles of the Declaration of Helsinki.

### 2.2 Clinical characteristics

Baseline demographic characteristics (age and sex), medical history (hypertension, diabetes mellitus, coronary heart disease, prior stroke or transient ischemic attack [TIA]), laboratory blood biomarkers, and transthoracic echocardiographic parameters (left atrial size and left ventricular ejection fraction) were obtained from the clinical records. Stroke risk was assessed by the CHA_2_DS_2_‐VASc score, which is calculated by adding the risk factors of congestive heart failure, hypertension, age 65–74 years or ≥75 years, diabetes mellitus, stroke or TIA, vascular disease, and female sex. Each parameter was weighted by “1,” except for stroke or TIA and age ≥75 years, which were weighted by “2.”^8^

### 2.3 Preparation for procedure

Antiarrhythmic medications were discontinued for at least five half‐lives before the ablation procedure. All patients received standard anticoagulation therapy for at least 1 month before the procedure. The international normalized ratio was maintained at 2–3 before the procedure in the patients receiving warfarin. A transesophageal echocardiography and computed tomography angiography (CTA) were performed before the procedure to exclude the presence of LA/left atrial appendage thrombus and to assess the anatomy of the left atrium (LA) and PV in detail.

### 2.4 PVI

All cryoablation procedures were performed under deep sedation using continuous infusion of midazolam and fentanyl. A single transseptal puncture was performed via a right femoral venous approach guided by fluoroscopy. After achieving LA access, 100 IU/kg heparin was administered to maintain an activated clotting time of ≥300 s. A 28‐mm cryoballoon (Arctic Front Advance; Medtronic Inc.) was then advanced in the LA via a steerable 15‐Fr sheath (FlexCath Advance; Medtronic). An inner lumen mapping catheter (Achieve; Medtronic) was then positioned in each PV ostium. Baseline electrical information was gathered for each PV ostium. The 28‐mm cryoballoon was advanced, inflated, and then positioned at each ostium. Optimal vessel occlusion was defined by selective contrast injection showing total contrast retention with no backflow into the LA. The ablation sequence consisted of treating the left superior PV (LSPV) first, followed by the left inferior PV (LIPV), right superior PV (RSPV), and right inferior PV (RIPV). When vessel occlusion was deemed satisfactory, delivery of cryoenergy to allow freezing was commenced. Two cryoballoon applications, each 3 min in duration, were applied for each PV. Successful PVI was defined as an absence of any PV potentials or their dissociation from any atrial activity. Adjustment of the application time and the necessity of additional freezing were left to the operator's discretion.^9^ If the nadir temperature of the balloon exceeded −55°C, the ablation would be terminated.

### 2.5 Electrophysiological characteristics of the SVC trigger

**Figure 1:**
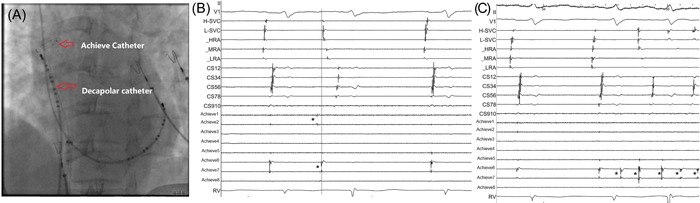
Spontaneous ectopic beats originating from the SVC. (A) Schematic diagram for SVC mapping. (B) A single atrial premature contraction originating from a spontaneous ectopic beat in the SVC. The sharp SVC potential (asterisk) recorded in the Achieve catheter was the earliest activation during firing in the SVC. This activation was then conducted from the SVC to the high right atrium according to the steerable decapolar catheter placed in the SVC. (C) Spontaneous ectopic beats (asterisk) originating in the SVC triggered and maintained atrial fibrillation. There were high‐frequency activations in the SVC. SVC, superior vena cava.

Atrial burst pacing and routine pharmacological testing with isoproterenol were performed to identify non‐PV triggers after PVI. If the AF remained after PVI, external electrical cardioversion was performed to restore normal SR. If AF was spontaneously reinduced after cardioversion, atrial pacing, or pharmacological testing with isoproterenol, the triggers were mapped and localized by integrating information from a 12‐lead electrocardiogram and the intracardiac activation patterns. Rapid firing in the SVC was first recorded by the steerable decapolar catheter and then mapped together with the Achieve catheter for all patients (Figure 1A). The sharp SVC potential recorded in the Achieve catheter was the earliest activation during firing in the SVC. The activation was then conducted from the SVC to the high right atrium (HRA) according to the steerable decapolar catheter placed in the SVC (Figure 1B,C). The SVC trigger was confirmed when at least one of the following criteria was met: (a) a far‐field blunt right atrial potential preceded the near‐field sharp SVC potential during normal SR and the activation sequence of the double potentials was reversed during initiation; (b) high‐frequency activations in the SVC that triggered and maintained AF were documented whereas the coronary sinus electrograms were more organized.

### 2.6 SVC isolation procedure

**Figure 2:**
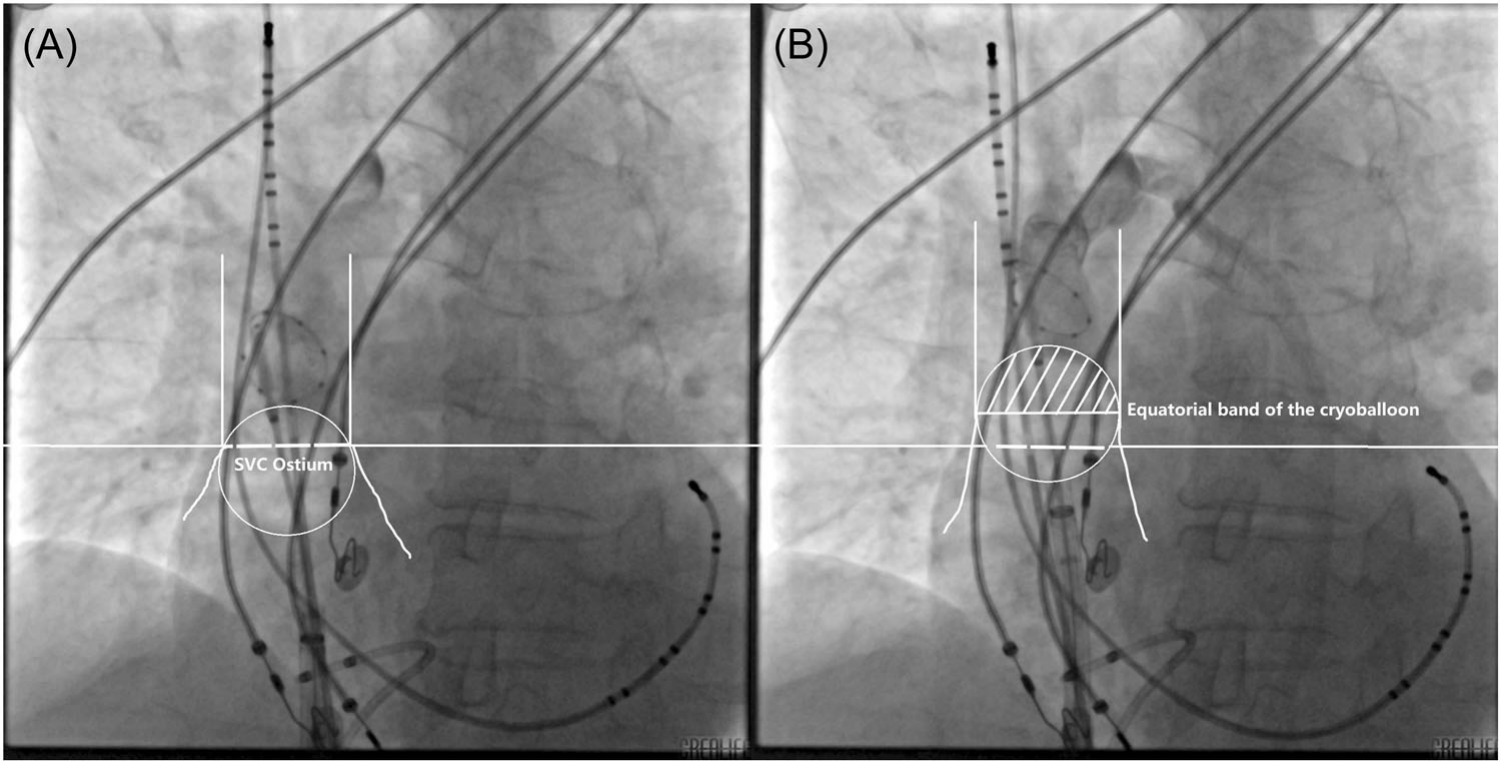
Isolation of the SVC using the cryoballoon. (A) The cryoballoon was inflated in the right atrium and advanced toward the junction of the SVC and right atrium. After total occlusion was confirmed by dye injection with total retention of contrast in the SVC, the SVC‐RA junction was determined. (B) The cryoballoon was deflated, advanced into SVC, then reinflated, and pulled back gently. The equatorial band of the cryoballoon was then set slightly above the SVC‐RA junction to ensure that the distance between the two was within approximately 5 mm. RA, right atrium; SVC, superior vena cava.

First, the cryoballoon was inflated in the RA and advanced toward the SVC‐RA junction. After total occlusion was confirmed by injection of dye with total retention of contrast in the SVC, the SVC‐RA junction was determined (Figure 2A).

Second, the cryoballoon was deflated, advanced into SVC, then reinflated, and pulled back gently. Due to the mechanical stress exerted on the muscular sleeve by the inflated cryoballoon in the SVC, spontaneous SVC ectopic beats or atrial tachycardia would terminate immediately in some cases, confirming the role of the SVC in triggering and maintaining AF. The equatorial band of the cryoballoon was then set slightly above the SVC‐RA junction. Using the steerable decapolar catheter (spacing 2‐5‐2 mm) as a scale label, the distance between the SVC‐RA junction and the equatorial band of the cryoballoon was confirmed to be within approximately 5 mm (Figure 2B).

**Figure 3:**
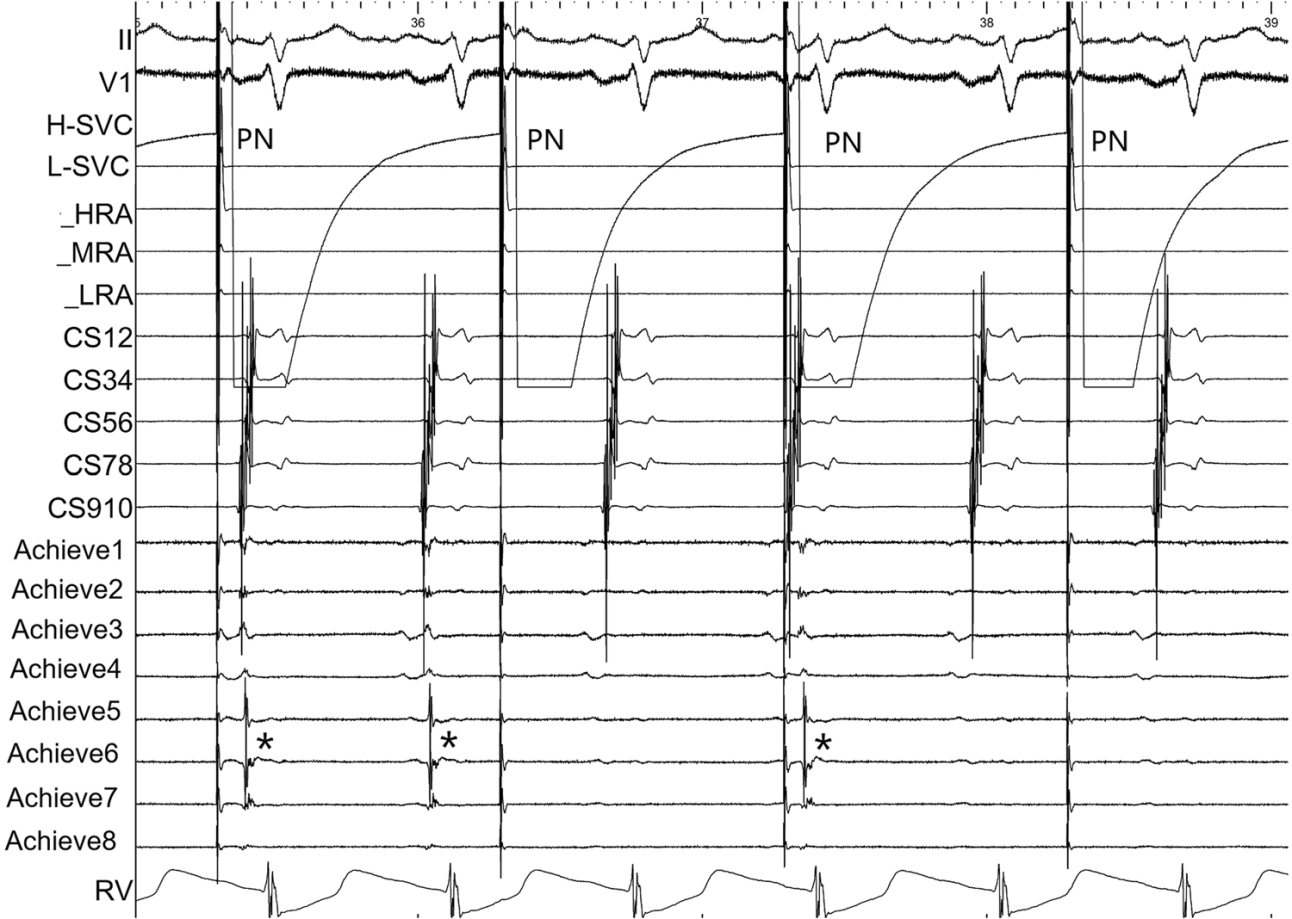
Isolation of SVC potentials. The SVC potentials (asterisk) were recorded by the Achieve catheter. The potentials. gradually became delayed, then started conduction at 2:1, and were finally successfully isolated. PN, phrenic nerve; SVC, superior vena cava.

The Achieve catheter was displaced in a backward manner to a more proximal position of the cryoballoon. Real‐time SVC potential was sought and recorded by the Achieve catheter or the steerable decapolar catheter in the SVC before the cryoablation. The venogram was repeated immediately before the cryoblation to confirm complete occlusion of SVC for each patient. Time to isolation (TTI) was determined by the complete disappearance of the SVC potential or its dissociation from electrical activity in the RA (Figure 3). SVC isolation was performed with a TTI plus 90‐s duration cryoenergy application. Adjustment of the application time and the need for additional freezing were left to the operator's discretion. If loss of PN capture, sinus bradycardia, junctional rhythm, or a nadir temperature of the balloon exceeding −55°C occurred, the application was terminated immediately.

After isolation of the SVC and a wait period of 20 min, routine pharmacological testing with isoproterenol was performed^10^ to check for dormant conduction. SVC‐RA entry block following SVC isolation was confirmed.

### 2.7 PN monitoring

Before ablation of the RSPV, RIPV, and the SVC, the steerable decapolar catheter was placed distally in the SVC and the diaphragm was stimulated by pacing the ipsilateral PN with a 1500‐ms cycle and a 20‐mA output. In this study, we monitored the excursion of the diaphragm by manually palpating the abdomen during pacing of the PN while ablating the right‐sided PVs and the SVC. The PN was continuously monitored to reduce the risk of paralysis of the PN.

### 2.8 Postprocedural management

All patients were continuously monitored by electrocardiographic telemetry after the procedure. Oral anticoagulation was prescribed for at least 2 months. The outpatient clinic visits were scheduled at 1, 3, and 6 months, and every 6 months thereafter. A 24‐h Holter monitoring system was the main method for recurrence assessment, which was performed at 1, 3, 6, and 12 months during follow‐up. Patients who developed symptoms that raised suspicion for recurrence of AF were also advised to undergo an electrocardiographic examination at the nearest hospital. Three months after the ablation procedure was considered to be the blanking period. Recurrence was defined as any documented atrial episodes with a duration longer than 30 s.

### 2.9 Statistical analysis

Continuous variables are expressed as the mean ± standard deviation and categorical variables as the percentage. The Student's *t*‐test was used to compare continuous variables. Categorical data were analyzed using the *χ* ^2^ test or Fisher's exact test as appropriate. The AF recurrence‐free survival rate was estimated by Kaplan–Meier analysis. All statistical analyses were performed using SAS statistical software version 9.2 (SAS Institute Inc.). A *p* value of <.05 was considered statistically significant for all determinations.

## 3 RESULTS

### 3.1 Baseline characteristics

A total of 650 consecutive patients with AF who were scheduled to undergo cryoballoon ablation at Shanghai Ruijin Hospital between July 2019 and July 2021 were screened. Forty‐three (6.62%, including six redo cases) of these patients were found to have AF originating from foci in the SVC during the procedure and were enrolled in the study. The SVC was electrically isolated by cryoballoon ablation after PVI. The study population had a mean age of 62.6 ± 9.3 years, and 60.5% were men. The baseline patient characteristics are shown in Table 1.

**Table 1:** Baseline demographic characteristics

Characteristics	*n* = 43
Age (years)	62.6 ± 9.3
Male gender (*n*, %)	26 (60.5%)
PAF (*n*, %)	33 (76.7%)
BMI (kg/m^2^)	24.4 ± 2.5
DM (*n*, %)	4 (9.3%)
Hypertension (*n*, %)	21 (48.8%)
Stroke/TIA	0
Coronary heart disease (*n*, %)	5 (11.6%)
Heart failure (*n*, %)	0
CHA_2_DS_2_‐VASc score	1.7 ± 1.1
LAD (mm)	40.6 ± 4.1
LAV (cm^3^)	115.9 ± 35.3
LVEDD (mm)	47.8 ± 4.1
LVEF (%)	65.7 ± 3.6
d‐dimer (mg/L)	0.27 ± 0.08

Abbreviations: BMI, body mass index; DM, diabetes mellitus; EF, ejection fraction; LAD, left atrial diameter; LAV, left atrial volume; LVEDD, left ventricular end‐diastolic diameter; LVEF, left ventricular ejection fraction; PAF, paroxysmal atrial fibrillation; TIA, transient ischemic attack.

### 3.2 Procedural parameters of PVI

A total of 183 PVs (1 left common PV, 42 LSPVs, 42 LIPVs, 43 RSPVs, 12 right middle PVs, and 43 RIPVs) were targeted in the 43 patients. A 28‐mm cryoballoon was applied in all cases and PVI was successfully achieved in all PVs. A mean 8.4 ± 2.6 cryoballoon freezes were applied per patient and 2.1 ± 0.8, 2.0 ± 0.8, 1.9 ± 1.0, 1.2 ± 0.4, and 2.1 ± 1.0 for the LSPV, LIPV, RSPV, right middle PV, and RIPV, respectively. There were no PN injuries during the ablation of the PV.

### 3.3 Electrophysiological characteristics of the SVC trigger

Recurrent spontaneous firing in the SVC that triggered AF was observed in 33 patients. AF resumed immediately after external electrical cardioversion in 10 patients. Therefore, SVC isolation was performed under AF. After the initiation of SVC cryoablation (10–30 s after the onset of cryoablation), seven patients with AF were terminated and converted to stable SR. For these patients, SVC is the trigger and driver of AF; the remaining three patients were treated with cryoablation for 30 s first to temporarily inhibit the SVC trigger activity, then electrical cardioversion was performed again. After stable SR was restored, cryoablation was continued. For these three patients, once AF persisted, the AF cycle length was longer in the SVC than in the atrium. This means that AF is triggered by SVC, but the substrate for AF perpetuation is in the atrium. Without 30 s of cryoballoon ablation pretreatment, just couple of beats coming from the SVC trigger foci could elicit new episode of AF immediately after electrical cardioversion. Rapid SVC potentials driving the AF were first recorded by the steerable decapolar catheter and then mapped together with the Achieve catheter in all patients.

### 3.4 Isolation of the SVC

The SVC was isolated in all patients, with no cases of reconnection during the 20‐min wait period. Interestingly, we also found that when the inflated balloon was inside the SVC, spontaneous ectopic beats or atrial tachycardia originating in the SVC terminated immediately in 16 patients (37.2%) by mechanical stress.

The equatorial band of the cryoballoon was set slightly (4.32 ± 0.71 mm) above the SVC‐RA junction for isolation of the SVC. The mean number of freeze cycles for SVC in each patient was 2.5 ± 1.4. Recording of real‐time potentials during isolation of the SVC was detected in all 43 patients. The mean TTI was 24.5 ± 10.7 s and the temperature during isolation of the SVC was −26.1 ± 6.3°C. The mean ablation time was 99.8 ± 22.7 s. The minimum temperature reached was −44.9 ± 5.5°C. The mean diameter of the SVC was 20.9 ± 0.9 mm. The procedure time was longer in patients who underwent isolation of the SVC than in those in whom isolation was not attempted (108.5 ± 26.8 vs. 85.4 ± 17.3 min; *p* < .001). There were also significant differences in the fluoroscopy time and dose for patients who did and did not undergo isolation of the SVC (fluoroscopy time: 18.8 ± 7.5 vs. 12.6 ± 4.5 min, *p* < .001; fluoroscopy dose: 420.0 ± 215.7 vs. 285.7 ± 187.0 mGy; *p* < .001). The procedural details are shown in Table 2. Dissociated electrical activity from the SVC was observed in 10 patients (23.3%) after the isolation procedure.

**Table 2:** Procedural details

	*n* = 43
SVC diameter, mm	20.9 ± 0.9
Mean total procedural time, min	108.5 ± 26.8
Mean fluoroscopy time, min	18.8 ± 7.5
Average radiation dose, μGycm^2^	4754.1 ± 2142.2
Average radiation dose, mGy	420.0 ± 215.7
Mean number of applications, *n*	2.5 ± 1.4
Mean ablation duration (s)	99.8 ± 22.7
Real‐time SVC potential recordings, *n* (%)	43(100.0%)
Time to isolation (s)	24.5 ± 10.7
Temperature at isolation, °C	−26.1 ± 6.3
Nadir temperature, °C	−44.9 ± 5.5

Abbreviation: SVC, superior vena cava.

### 3.5 Safety and complications

During the isolation procedure, one patient (2.33%) developed a transient PN injury. The activity of the PN returned to normal before the end of the procedure. Interruption of the application instantaneously led to the complete resumption of PN activity. No patient sustained an SN injury (sinus bradycardia and junction rhythm). All patients tolerated occlusion of the SVC during the procedure without any significant hemodynamic or clinical impact. No changes in oxygen saturation were found. There were no minor or major procedure‐related complications, including no access site problems or persistent paralysis of the PN.

### 3.6 Follow‐up

Considering the blanking period of 3 months, freedom from AF rates at 6 and 12 months was 97.7% and 93.0% without the use of antiarrhythmic agents, respectively. The survival curve can be found in the Supplement. Recurrence manifested as AF in two cases and as atypical atrial flutter in one. Remarkably, in two patients who underwent a repeat procedure, durable PV isolation was documented, and the SVC was still isolated. No stenosis of the SVC was documented in the patients who underwent a repeat ablation procedure. No sinus bradycardia or sinus arrest was observed in any patient during follow‐up. No patient had signs or symptoms of SVC stenosis, such as dyspnea, facial or upper extremity congestion.

## 4 DISCUSSION

To the best of our knowledge, this is the first study to assessed the safety and efficacy of isolation of the SVC using the novel cryoballoon method. The study had two main findings. First, isolation of the SVC using this novel method was effective. Second, isolation of the SVC using this method could avoid injury to the SN.

The SVC is a well‐known trigger for AF. Embryologically, the SVC arises from communication between the sinus venosus and the RA. Structures that originate from the sinus venosus have demonstrated an ability to express cells that retain ectopic pacing ability and can become a focus of arrhythmia.^11^ These specialized cells have been found to reside within large atrial myocardial extensions (or myocardial sleeves) on the SVC and serve as independent triggers for arrhythmias.^12^ These myocardial sleeves extend for an average of 13.7 mm into the SVC and have a mean thickness of 1.2 mm.^12^ Therefore, the SVC is an important target during AF ablation, and empirical isolation of the SVC using radiofrequency energy has been confirmed to be beneficial in patients with AF.^4^

Compared with RFA, cryothermal ablation results in a more homogenous border zone.^13^ Cryoballoon ablation can create a circumferential lesion at the PV ostium with a more convenient single shot. Several studies have reported the feasibility of SVC isolation using cryoballoon ablation. However, whether this novel method could facilitate SVC isolation and improve the safety of the procedure had been unclear.

In the present study, the SVC was successfully isolated using a second‐generation cryoballoon in all patients. Freedom from AF rates at 6 and 12 months was 97.7% and 93.0% without use of antiarrhythmic agents, respectively. Therefore, this novel method for electrical isolation of the SVC is effective.

The SVC‐RA junction is a complicated anatomical area with multiple structures located in close proximity, including the SN and right PN. Complications, such as SN or PN injury and stenosis of the SVC have been observed in patients who undergo RFA of the SVC.^14^, ^15^

Injury to the right PN is the major concern and is one of the most common complications associated with the use of the cryoballoon ablation technique.^16^ During RFA of the SVC, especially of the posterolateral portion of the vessel, ablation should be avoided if the PN could be captured by high‐output pacing at the ablation site. However, most procedures require radiofrequency delivery to that area to complete the electrical isolation of the SVC, and this might also be a nonspecific predictor of PN injury due to the dependence on contact force and power.^17^, ^18^

Different techniques, such as palpation of the diaphragmatic excursion, monitoring of diaphragmatic compound motor action potentials, and the “double stop” strategy, have been proposed to avoid injury to the PN.^19^ In this study, we monitored the excursion of the diaphragm by manually palpating the abdomen during pacing of the PN while ablating the right‐sided PVs and the SVC. Transient PN injury was observed in one patient (2.33%), and complete recovery of diaphragmatic contraction was observed before the end of the procedure.

Damage to the SN has been described as a potential complication of SVC isolation. There have been reports of permanent SN injury requiring pacemaker implantation during isolation of the SVC by RFA.^20^, ^21^ The explanations put forward for these injuries were damage to the sinoatrial node artery and irreversible radiofrequency thermal energy. The upper border of the SN is located at the anterolateral SVC‐RA junction; therefore, ablation should be performed slightly above the junction and slightly into the SVC.^15^ For the second‐generation cryoballoon, the zone of optimal cooling comprises the whole frontal hemisphere.^22^ In the present study, the equatorial band of the cryoballoon was set 4.32 ± 0.71 mm above the SVC‐RA junction. Therefore, no SN injuries were encountered in this study. Previous studies^1^, ^6^, ^7^ using cryoballoon for SVC isolation were performed as follows: the cryoballoon was inflated in the RA and advanced toward the SVC ostium to occlude the vessel. After total occlusion was confirmed by dye injection with total retention of contrast in the SVC, cryoenergy application was started. Thus, the equatorial zone of the cryoballoon might be located below the SVC‐RA junction when cryoenergy application was started. Transient SN injury was observed in the study published by Wei et al.^6^ using this method.

Stenosis of the SVC has also been reported during RFA.^23^ However, in clinical practice, the incidence of clinically significant SVC stenosis is very low. Callans et al.^24^ reported that RFA for inappropriate sinus tachycardia in the human SVC resulted in an average acute luminal narrowing of 24%. However, no patients in their study developed clinical symptoms of SVC syndrome. Cryoablation could be performed as a strategy to reduce the risk of stenosis of the SVC, possible because of the different mechanisms via which radiofrequency thermal energy and cryoenergy create lesions. No acute or chronic SVC stenosis was observed during follow‐up in the present study.

The optimal dosing strategy for the isolation of the SVC remains unknown. For PVI, previous studies have reported that a single 3‐min technique is highly effective in achieving a favorable outcome.^25^ Furthermore, a TTI‐guided strategy has been confirmed to be effective.^26^ A recent study in an animal model found that a 90‐s dosing strategy isolated the SVC effectively.^27^ In the present study, real‐time SVC potential was recorded in all 43 patients, and the cryotherapy protocol of TTI plus 90 s was feasible with a favorable outcome. The mean values for TTI and ablation time were 24.5 ± 10.7 and 99.8 ± 22.7 s, respectively, which is consistent with those in a previous report.^6^ Interestingly, we also found that spontaneous SVC ectopic beats or atrial tachycardia terminated immediately in 16 patients (37.2%) when the balloon was inflated inside the SVC, which was attributed to the mechanical stress exerted on the muscular sleeve by the cryoballoon. This phenomenon indicates that the electrical connection between the RA and the SVC is poorer than that between the LA and PV. Therefore, we postulate that the cryotherapy strategy of TTI plus 90 s might be feasible for isolation of the SVC.

## 5 LIMITATIONS

This study has some limitations that must be considered in future analyses. First, the study had a single‐center design with a relatively small sample size. Further randomized studies in large samples are needed to confirm our findings. Second, stenosis of the SVC was not actively investigated. Nevertheless, no patient in the study experienced any symptom potentially related to stenosis of the SVC during follow‐up. Third, this is a pure two‐dimensional and anatomical‐based technique, although there was no SN injury during the procedure, in further study, activation mapping could be performed during SR to localize the actual site of the SN using three‐dimensional mapping system. Our study findings require consolidation in a large prospective study.

## 6 CONCLUSION

This novel method for isolation of the SVC using a second‐generation 28‐mm cryoballoon is safe and feasible when the SVC driver during AF is determined. This method could effectively avoid SN injury. However, the function of the PN should be carefully monitored during an SVC isolation procedure.

## CONFLICT OF INTEREST

The authors declare no conflict of interest.

## Supporting information

Supplementary information.

## Data Availability

The data of this study are available from the corresponding author upon request
