# Supplementary material for: Initial experience of a novel method for electrical isolation of the superior vena cava using cryoballoon in patients with atrial fibrillation
Source: Clin Cardiol. 2022 Nov 20;46(2):126–33. doi: 10.1002/clc.23947 (PMC9933103; doi:10.1002/clc.23947)

**Supplemental Appendix**

Kaplan‐Meier curve showing freedom from atrial fibrillation (AF) after the procedure. Freedom from AF rates at 6 and 12 months was 97.7% and 93.0% without use of antiarrhythmic agents, respectively.


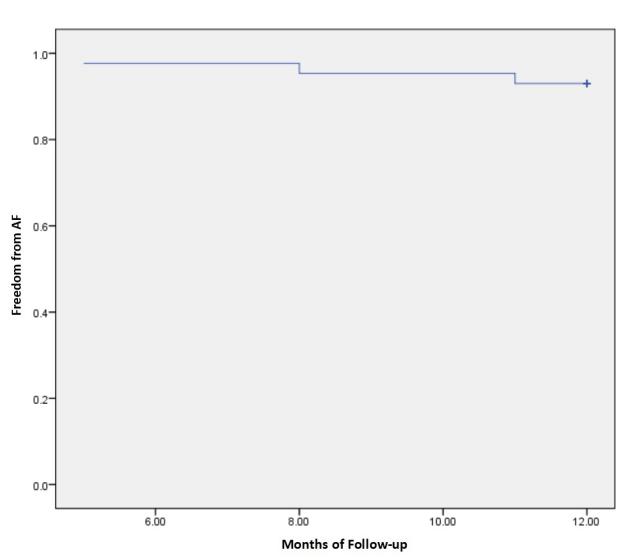

Supplement: Supplementary file 1 — Supplementary information. [file CLC-46-126-s001.docx]
